# Antibiotics-Free Compounds for Chronic Wound Healing

**DOI:** 10.3390/pharmaceutics14051021

**Published:** 2022-05-09

**Authors:** David O. Oluwole, Lucy Coleman, William Buchanan, Tao Chen, Roberto M. La Ragione, Lian X. Liu

**Affiliations:** 1 Chemical and Process Engineering Department, Faculty of Engineering and Physical Science, University of Surrey, Guildford GU2 7XH, UK; l.coleman@surrey.ac.uk (L.C.); t.chen@surrey.ac.uk (T.C.); 2 Phytoceutical Limited, Midhurst, West Sussex GU29 9DJ, UK; will@phytoceutical.co.uk; 3 School of Biosciences and Medicine, Faculty of Health and Medical Sciences, University of Surrey, Guildford GU2 7XH, UK; r.laragione@surrey.ac.uk; 4 School of Veterinary Medicine, Faculty of Health and Medical Sciences, University of Surrey, Guildford GU2 7AL, UK

**Keywords:** antimicrobial, anti-inflammatory, antibiotics-free, antimicrobial resistance, chronic wounds

## Abstract

The rapid rise in the health burden associated with chronic wounds is of great concern to policymakers, academia, and industry. This could be attributed to the devastating implications of this condition, and specifically, chronic wounds which have been linked to invasive microbial infections affecting patients’ quality of life. Unfortunately, antibiotics are not always helpful due to their poor penetration of bacterial biofilms and the emergence of antimicrobial resistance. Hence, there is an urgent need to explore antibiotics-free compounds/formulations with proven or potential antimicrobial, anti-inflammatory, antioxidant, and wound healing efficacy. The mechanism of antibiotics-free compounds is thought to include the disruption of the bacteria cell structure, preventing cell division, membrane porins, motility, and the formation of a biofilm. Furthermore, some of these compounds foster tissue regeneration by modulating growth factor expression. In this review article, the focus is placed on a number of non-antibiotic compounds possessing some of the aforementioned pharmacological and physiological activities. Specific interest is given to *Aloe* *vera*, curcumin, cinnamaldehyde, polyhexanide, retinoids, ascorbate, tocochromanols, and chitosan. These compounds (when alone or in formulation with other biologically active molecules) could be a dependable alternative in the management or prevention of chronic wounds.

## 1. Introduction

Skin wounds are injuries that compromise or damage the structural integrity and optimal functioning of the skin and can be grouped into acute and chronic depending on the skin repair and recovery timeframe. Acute wounds (AWs) can range from skin surface scratches to deep injuries, with skin repair and recovery achieved following the normal healing process, which is usually attained within 3 weeks. At AW sites, the body quickly initiates rapid cell migration, including fibroblast and keratinocytes, whilst modulating suitable degrees of inflammation, innervation, and angiogenesis. Chronic wounds (CWs) include diabetic foot ulcers, pressure sores, venous leg ulcers, arterial ulcers, and ischemic and surgical wounds requiring medical interventions for their management. CWs do not follow the same cellular and molecular sequence as normal AWs [1,2,3,4].

Wound care continues to gain tremendous attention worldwide, both in academia and industry, and this is attributed to its significance as wounds contribute to major health burdens [1,5,6,7]. Globally, nearly 2% of hospitalised patients suffer from chronic wounds, and this is common in adults with advanced age, which often affects the wound recovery timeframe due to many factors, including immune suppression [6]. In the UK, the NHS annual cost for wound management was over £5.6 billion in 2017/2018 [5]. In the USA, 8.2 million Medicare beneficiaries suffer from chronic wounds or similar health burdens, costing over £24 billion in 2014, underscoring the magnitude of this healthcare challenge [1,7]. Moreover, the management costs for diabetic foot ulcers and surgical wounds are exorbitant compared to those of other chronic wounds [1].

Several modalities have been recommended for wound care, including wound dressing, surgery, hyperbaric oxygen, and antibiotics. In most cases, antibiotics are employed to reduce the bacteria loads around the wound site and are sometimes used in combination with wound dressings, which can foster efficient inflammation and proliferation phases. In addition, the debridement of the wound site is sometimes effective and, in a worst-case scenario, a surgical procedure is conducted on the affected region of the body [2,8]. The skin performs essential roles in the body, including protection against external attacks, such as invasive microbial infections [5]. Wound infection is known to be dependent on several factors, including a patient’s immune system, the virulence of the microbe involved, and the nature of the wound [9,10,11,12]. This review focuses on the chronic wound healing properties of antibiotics-free compounds, including *Aloe vera*, curcumin, cinnamaldehyde, polyhexanide, retinoids, ascorbate, tocochromanols, and chitosan. Literature data on the anti-inflammatory, antimicrobial, and wound healing efficacy of these compounds were considered when alone or in formulation with other biologically active molecules.

## 2. Chronic Wounds—Formation and Antimicrobial Resistance (AMR)

The chronicity of wounds is affected by several contributory factors, including hormonal imbalances, cytokines, and growth factors. More importantly, bacterial infections have been implicated as the predominant feature in most chronic wound microenvironments, including *Staphylococcus aureus* and *Pseudomonas aeruginosa* [10]. These bacteria exist in polymicrobial forms forming biofilms that afford them protection against the host’s immunity and conventional antibiotics. *S. aureus* biofilms are sometimes present close to the surface of CWs, while *P. aeruginosa* biofilms appear deep within wound tissue [9,10]. The recalcitrant disposition of these microbes has been implicated as one of the causalities of antimicrobial resistance [13].

### 2.1. Wound Healing Process

**Figure 1:**
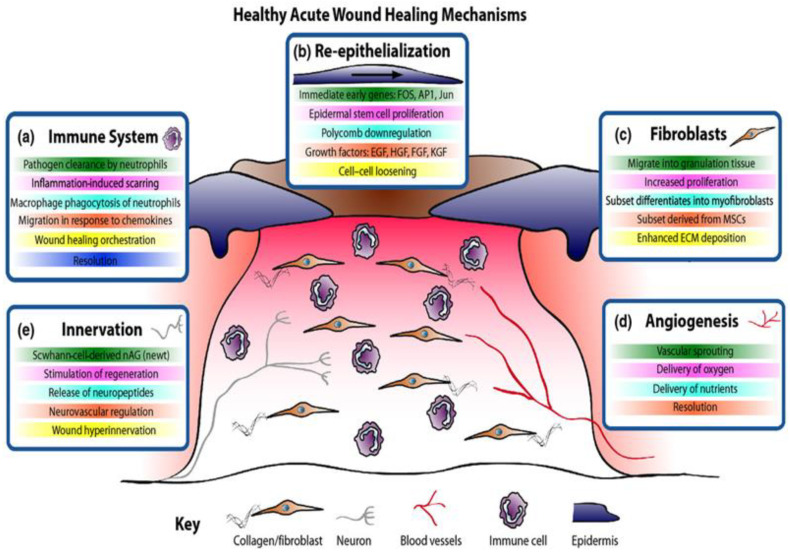
Wound healing process of healthy acute wounds as shown by Martin and Nunan [2]. Healthy AWs adhere to well-modulated cellular and molecular events, resulting in the rapid clearance of invasive microbes and subsequent removal of apoptotic neutrophils, with regulated cell migration promoting early wound contraction and tissue remodelling [2].

The skin is known to afford a variety of important protective functions; however, whenever its integrity is compromised by injuries, the body initiates a dynamic process at the wound site, resulting in the recovery of the tissue and restoration of the skin’s barrier function [14]. Four sequential unique phases are known to be involved in healthy acute wound healing, including haemostasis, inflammation, proliferation, and remodelling, as shown in Figure 1 [2]. The haemostasis phase is often observed on injury occurrence, and it is composed of platelet aggregation, leading to the formation of blood clots [14,15]. 

**Figure 2:**
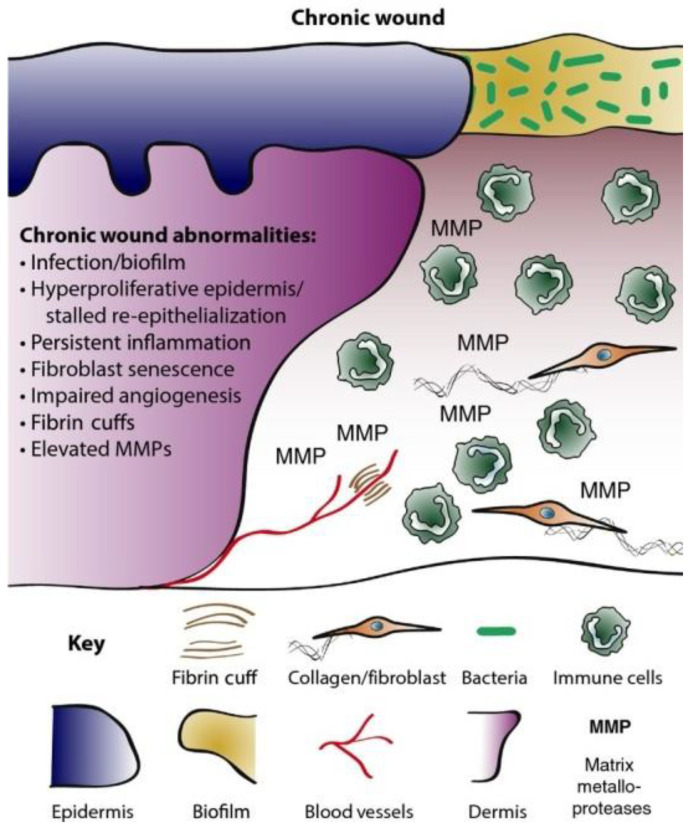
Biology of chronic wounds as shown by Martin and Nunan [2]. CWs are usually influenced by microbial infections resulting in persistent inflammation due to the recruitment of highly inflammatory infiltrates and inhibition of tissue regeneration [2].

This is regarded as the body’s response to protecting its structure by regulating bleeding and fostering the process of wound healing. However, this phase can be impaired if the patient is suffering from underlying medical conditions, such as diabetes and cancer. This is followed by the inflammation stage, which promotes the movement of blood cells (including phagocytic neutrophils and macrophages) to the wound site to afford protection to this site [15]. In this phase, extraneous particles (such as invasive microbes) are initially removed by the phagocytic neutrophils and the macrophages eliminate the dead neutrophils while fostering the rapid closure of the wound [2,14,16]. In the proliferative stage, the re-epithelialisation of wounds commences within hours of the injury’s occurrence [14]. This is followed by the formation of new blood vessels, including angiogenesis or neovascularisation, and the re-establishment of the perfusion to sustain the newly formed tissues [16]. Afterwards, the generation and deposition of fragments of the extracellular matrix (ECM), including collagen fibres and granulation tissues, is initiated [14,15]. The final phase involves tissue remodelling and scar tissue formation [14,15]. Moreover, when the healthy acute wounds fail to adhere to the standard healing time course, it often leads to chronic wounds (ulcerative skin defect) or hypertrophic scars (excessive scar tissue formation), as illustrated by Martin and Nunan [2] in Figure 2. Healthy acute wounds initiate appropriate modulation of the cellular and molecular sequence required to promote wound healing within a standard time course. On the other hand, the modulation of cellular and molecular events in chronic wound is often disrupted by a number of factors, including invasive microbial infection leading to high infiltration of inflammatory cells, including neutrophils, and these cells could be phenotypically non-identical to their corresponding analogues in normal acute wound healing. This is further complicated by persistent inflammation, unlike AW healing, which initiates well-modulated inflammatory response resolution. Moreover, the proliferation of keratinocyte gene expression in chronic wounds is partially activated with deteriorating fibroblasts having a reduced migratory magnitude [2]. 

Efficacious wound care agents are expected to protect wound tissues from bacterial infection, modulate inflammation, and scavenge free radicals (antioxidant), as well as foster cell migration and growth to aid in the recovery of damaged tissues [17].

### 2.2. Chronic Wounds

A wound is considered to be chronic when it fails to undergo the normal phase for the recovery and restoration of the structural and functional integrity within 3 months (Figure 2) [2]. A CW’s microenvironment is characterised by the presence of dead tissue, and high influx of metalloproteases and pH [2,4,10]. The pH of CWs is known to be alkaline, as opposed to those of the normal skin and AW environment, which are slightly acidic [18]. This pH change is often attributed to the presence of high bacteria loads, as an alkaline pH is capable of breeding or encouraging the proliferation of bacteria in wounds [18,19]. A slightly acidic pH has been shown to foster wound recovery, and this could be one of the contributing factors in rapid AW recovery, as they are devoid of high bacteria loads [18,19]. Reports by Gray et al., James et al., and Schierle et al. suggested that the CW environment is capable of fostering the development of high bacteria loads and biofilms, which are sometimes recalcitrant to antibiotics [19,20,21]. This causality has been attributed to the delay in the re–epithelisation of compromised or defective skin [20]. A number of studies have demonstrated that almost 60% of CWs possess biofilms, representing an almost 10-fold higher association in comparison to AWs [22,23]. Moreover, numerous reports have shown that the CW microbial environment exists as polymicrobial with the presence of multiple pathogenic Gram-positive and Gram-negative bacteria (*Pseudomonas aeruginosa*, *Escherichia coli*, and *Staphylococcus aureus*), and these are potential biofilm formers that often account for antimicrobial resistance (AMR) [13,19,24,25,26,27,28]. These biofilms tend to change the properties of bacteria through their extracellular polysaccharide matrix (EPS), which affords them a protective shield against host immunity and chemotherapeutic intervention, including antibiotics. This is often achieved not only by inactivating antibiotics, but also by the regulation of the pH and metabolic state of the polymicrobial environment, contributing to the virulence of the bacteria [24,25,26,27,28,29]. 

Microbial colonisation and growth resulting in wound infections have been reported to be a contributory factor to delayed wound healing, which can sometimes lead to devastating effects, including low work productivity, lengthy hospitalisation, amputation, and death [29,30]. This influences the proper functioning of keratinocytes and fibroblasts (skin cells) by impairing the inflammation phase of wounds, and this is one of the leading causatives that influence the chronic wound environment [14,19,20,31]. Moreover, factors such as an inefficient blood supply at the wound site and inappropriate treatment modalities have been reported to cause non–healing wounds [8,9,10]. Some of these infections do not only occur in community setting, but studies have also shown that there has been an alarming increase in the number of bacterial infections occurring in healthcare facilities. These bacteria mostly infect their hosts through the skin and respiratory tract in contaminated hospital environments or through contaminated food. Moreover, infections can occur through the use of contaminated medical devices, including catheters and joint prostheses [25].

### 2.3. AMR in Chronic Wounds

Globally, WHO data suggest that AMR accounts for significant morbidity and circa 700,000 mortalities annually [32]. With the rapid rise of AMR contributing to the increased burden of wound care to the health care community [33,34], it is imperative to explore antibiotics-free compounds or their combination with antibiotics in the management or elimination of chronic wounds. Wounds infected by bacteria, including those capable of forming biofilms, are known to be recalcitrant to host defence and antibiotics, leading to AMR. The CW microenvironment is a suitable breeding site for bacterial colonisation and proliferation due to its relatively alkaline nature. This has given rise to AMR bacteria as a result of their biofilms that encapsulate the bacteria, affording them more defence against antibiotics. AMR prevalence is often associated with suboptimal administration of antibiotics, target modification, efflux mechanism, misuse, and the over-prescription of antibiotic agents [32,33,34]. 

**Figure 3:**
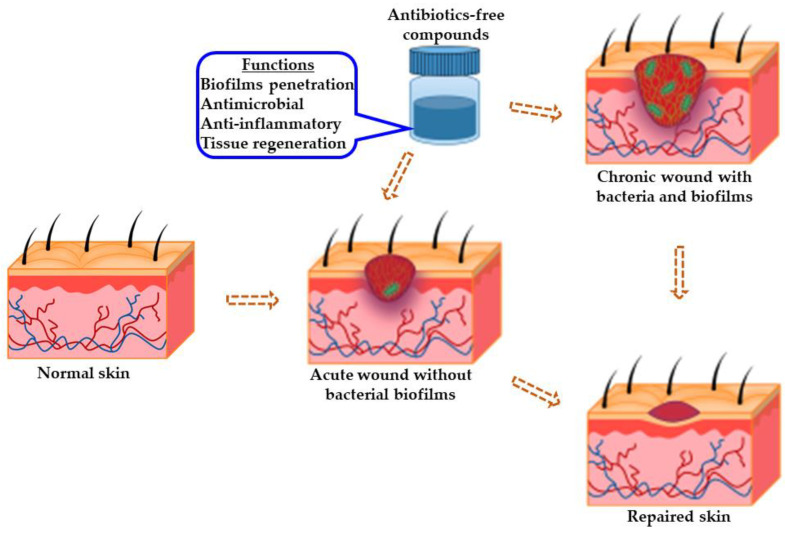
Schematic diagram showing the functionalities of antibiotics-free compounds towards wound healing. The morphology of healthy skin is distinct from that of chronic wounds due to defects in CWs’ skin anatomy and high invasive bacteria loads. Antibiotics-free compounds can serve as prophylactic or chronic wound care agents when alone or in formulation with antibiotics.

Recent studies have shown that antibiotics-free compounds could be a dependable and reliable strategy in the management of wounds, and this could be due to their mode of action, which is unaffected by AMR, unlike antibiotics, which have been shown to sometimes suffer from AMR, including methicillin-resistant *Staphylococcus aureus* (MRSA) [33,34,35,36,37]. Interestingly, these antibiotics-free compounds can elicit ideal pharmacological responses when independently administered with minimal or no adverse reaction. Specifically, they are active in the disruption of bacterial biofilms by attacking the EPS, known to afford support to the structural integrity of these biofilms. In addition, they can be employed as pharmaceutical or cosmetical adjuvants with other compounds, including (natural or semi-) synthetic antibiotics [38]. The functionalities of antibiotics-free compounds in wound healing are illustrated in Figure 3. 

The classification of antibiotics is based on their mode of action such as the inhibition of bacterial cell wall synthesis, protein production, DNA replication, and folic acid metabolism [39,40]. Antibiotics including penicillin, cephalosporins, carbapenems, and vancomycin function by disrupting bacteria cell wall production [39,40]. Protein biosynthesis inhibitors attack the 30 s or 50 s (spike) subunits of bacterial ribosomes, thereby preventing the production of bacterial proteins. Drug molecules in this class include chlortetracycline, tetracycline, doxycycline, chloramphenicol, and linezolid [39,40]. Quinolones (fluoroquinolones) [39,40] work by inhibiting bacterial DNA replication. The folic acid (FA) metabolism inhibitors, including sulphonamides and trimethoprim, work in synergy with the inhibition of the bacteria production pathway, with each drug disrupting each stage of FA metabolism [39,40].

In addition, the antimicrobial mechanism of action of antibiotics-free compounds is non-specific, except for the postulations that the phenolic compounds in plant materials are responsible for their antimicrobial activity [41]. Phenolics are the most prominent constituents of plant materials implicated in antibacterial activity. These phenolics include ketones, aliphatic alcohols, terpenes, isoflavonoids, aldehydes, and acids [41]. These compounds act by interacting with the bacterial cell structure, thereby disrupting the membrane functionality, leading to the deformation of the bacterial cell structure [38]. Moreover, the activities of these compounds are dependent on the administered concentrations, with minimal concentrations impairing the bacterium enzymatic functions, while a high dose is known to destroy bacterial proteins and also inhibit the bacterial metabolic pathway. Furthermore, some of these phenols work in synergy, which can influence their antibacterial activity in the disruption of bacterial peptidoglycans and the outermost membrane composed of lipopolysaccharides and proteins [38,41]. For instance, the antimicrobial mechanism of action of cinnamaldehyde (cinnamon) has been proposed to exhibit bacteriostatic or bactericidal actions by altering the bacterial cell membrane, preventing cell division, membrane porins, motility, and the formation of the biofilm [29,38].

Antibiotics-free compounds not only have the potential to reduce the invasive bacterial load and persistent inflammation of chronic wounds, but also modulate growth factor expression, which is necessary for tissue regeneration at the wound’s site, Figure 3.

## 3. Natural and Semi-Synthetic Compounds for Wound Healing 

The management of the microbial infection bioburden and tissue remodelling is a crucial aspect of wound care. Compounds with wound healing properties are effective under certain conditions, including low concentrations [42,43]; however, a lethal concentration has been reported to have safety concerns, ranging from prooxidant effects to DNA damage [43,44].

### 3.1. Curcumin

**Figure 4:**
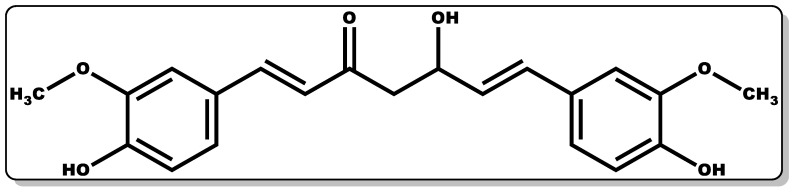
Molecular structure of (1E,6E)–1,7–Bis(4–hydroxy–3–methoxyphenyl)hepta–1,6–diene–3,5–dione (Curcumin).

Curcumin (Figure 4) is a lipophilic, bioactive compound obtained from the rhizome of the *Curcuma longa* Linnean plant, [45]. It is a phenolic dye with a bright yellow colouration constituting the major component of the curcuminoid of turmeric (*Curcuma longa*), accounting for the yellow colouration observed in turmeric [45]. Traditionally, it is used as an adjuvant (E100) in the food industry as a colouring and flavouring agent [45]. Beyond its traditional applications, the polyphenolic component of curcumin is known to actively regulate several signalling pathways and elicit a broad range of pharmacological activities [46], including anti-inflammatory [47,48,49], antioxidant [47,50], anticancer [51,52], antidiabetic [53], antiviral [54,55], and antibacterial activities [36,56,57,58,59,60,61]. Additionally, curcumin has been explored in the management of skin diseases, including psoriasis [62,63]. 

Curcumin possesses sparing to low aqueous solubility and poor stability, and this has limited its broad applicability when administered alone [64]. Moreover, the stability of curcumin is pH-dependent, with pH 7 to 8 accounting for about 90% of its degradation and a slightly acidic pH of 3 to 6.5 affording better stability in comparison to pH 7 to 8 [65]. Interestingly, some of its metabolites have been demonstrated to possess fascinating pharmacological dispositions, including anti-inflammatory, antimicrobial, anticancer, and cardioprotective properties [47,51,66]. The incorporation of curcumin with nanomaterials, micelles, and their micronised forms has been reported to exhibit improved solubility compared to it pristine form [67,68]. 

In vitro data have demonstrated the antibacterial and wound healing potential of curcumin [60,61,69]. In vitro study data of curcumin against *Escherichia coli* and *Bacillus subtilis* FtsZ demonstrated significant efficacy [60,61]. Comotto and co-workers explored curcumin in combination with t–resveratrol in the fabrication of an alginate-based breathable hydrogel dressing for the treatment of infected wounds; this combination was found to exhibit pivotal bactericidal activity [69]. The rising preclinical data on curcumin’s medicinal disposition have endeared the interest of researchers both in academia and industry to the exploration of its potential clinical administration in the management of diverse disease conditions [42,48,56,66]. The potential of curcumin in wound care was evaluated using various models, including rats, and curcumin was shown to be instrumental in improving epithelialisation, fibroblast proliferation, vascular density, collagen deposition, and reorganisation [70,71,72,73,74]. This was demonstrated in a study by Mehrabani et al. where curcumin was shown to foster wound healing by quenching free radicals and the subsequent modulation of inflammation through the inhibition of nuclear factor-B. Furthermore, it accelerates the regulation of collagen deposition and fibroblast migration by inducing transforming growth factor-β and stimulating angiogenesis and extracellular matrix accumulation, which are essential for tissue regeneration [70]. In another study by Miah et al., curcumin was applied to surgical wounds of Bengal goats, and it showed better wound recovery compared to the untreated groups [73]. The combination of curcumin in formulation with other molecules has proven to be advantageous in improving its solubility and efficacy. A recent study by Schiborr and co-workers demonstrated the improved solubility of curcumin when it was incorporated with polysorbate [68]. Moreover, enhanced antibacterial and wound care efficacy of curcumin has been reported when combined with hyaluronic or t-resveratrol [69,72,75]. This was shown in a study conducted by Sharma et al., where curcumin combined with hyaluronic acid was tested against bacteria and diabetic mice, and it exhibited bactericidal activity and rapid wound healing efficacy when compared with the untreated groups [72]. 

The anti-inflammatory mechanism of action (MOA) of curcumin could be due to its regulation of the gene expression of inflammatory cytokines capable of releasing high influxes of tumour necrosis factor (TNF), interleukin-6 (IL-6), and nitric oxide (NO), which could cause persistent inflammation [66]. Moreover, its non-specific antimicrobial MOA against bacteria likely works by binding to the FtsZ proteins, leading to the inhibition of the FtsZ protofilaments assembly, thereby suppressing bacterial growth and proliferation. In addition, its mode of action may be attributed to its disruption of the mecA gene transcription, resulting in a decrease in penicillin-binding protein-2α expression. For instance, this is demonstrated when curcumin binds with the peptidoglycan on the *S. aureus* cell wall, making it unavailable for the production of the new peptidoglycan, affecting the strength of the peptidoglycan layer, and triggering the breakdown of the bacterium [42,56]. The MIC (values in bracket, Table 1) of curcumin against *Staphylococcus aureus*, *Porphyromonas gingivalis*, *Escherichia coli*, *Staphylococcus epidermidis*, *Pseudomonas aeruginosa*, *Streptococcus mutans*, *Proteus mirabilis*, *Serratia marcescens*, and *Bacillus subtilis* [42] is summarised in Table 1.

### 3.2. Poly(hexamethylene biguanide) 

**Figure 5:**
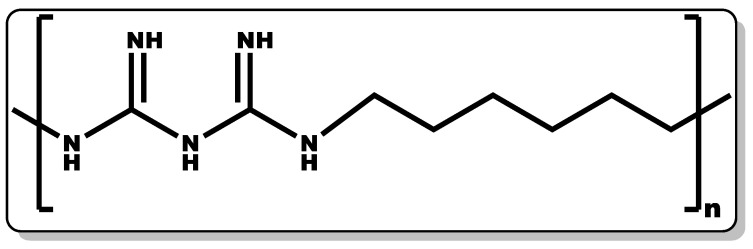
Structure of poly(hexamethylene biguanide).

PHMB (polyhexanide, Figure 5) is a known antiseptic with proven activity in the management of microbial infections. An increase in the polymer chain length of PHMB often leads to an improvement in its antimicrobial activity, and this structural chain length can be repeated 2 to 30 times [21,77]. PHMB has demonstrated high efficacy over a broad spectrum of microbes, including certain viruses [78,79], Gram-positive and Gram-negative bacteria [28,80], fungi [28,81], and certain parasites, particularly *Acanthamoeba* [82,83]. Its application has evolved beyond its traditional use as a multi-purpose disinfectant and deodoriser. Recent application of PHMB has been demonstrated in cosmetics and personal hygiene products as preservatives, with a concentration limited to 0.1% [43]. It is a synthetic polymer composed of a biguanide and hexamethylene moieties with structural similarity to naturally occurring antimicrobial peptides, giving it ease of penetrating bacterial cell membranes and eliciting bactericidal activity [84]. 

Specifically, it is known to mainly target the outer and cytoplasmic membranes. PHMB binds to the DNA and other nucleic acids of the cell membrane, leading to the destruction or inactivation of the bacterial DNA [21,85]. There is growing evidence of its wound healing efficacy when alone and incorporated in wound care products, including cleansing solutions, hydrogels, and dressings [21]. Preclinical data have suggested that PHMB possesses efficacy against wound–colonising bacteria, including MRSA and other pathogenic bacteria [86]. The PHMB MIC values against various pathogenic bacteria are listed in Table 1 [76]. 

Numerous studies have shown PHMB’s therapeutic activity in wound care management [12,87]. Wound-care products containing PHMB were found to exhibit anti-inflammatory dispositions by decreasing wound pain and malodour [12,87,88]. Moreover, it increases keratinocyte and fibroblast activity with improvement in granulation tissue formation and the elimination of dead tissues in the wound [12,89,90]. In a study by Lenselink and co-workers, 28 volunteers with critically colonised wounds were recruited and placed on PHMB-containing formulations. An increase in tissue granulation was observed within 24 weeks, and this was attributed to the antioxidant, anti-inflammatory, and antibacterial efficacy of PHMB [91]. In another study by Elzinga and co-workers, the tolerability and healing efficacy of PHMB were evaluated, and it was demonstrated to be well-tolerated and afford pain-free wounds with a good recovery timeframe [92]. However, PHMB has been reported to have detrimental effects at high concentrations, including fever and a generalised exanthema, which is thought to be the promotion of high nitric oxide by PHMB [93]. According to the ECHA, 0.1% PHMB is considered safe for application in cosmetics formulations [43].

### 3.3. Vitamin A

**Figure 6:**
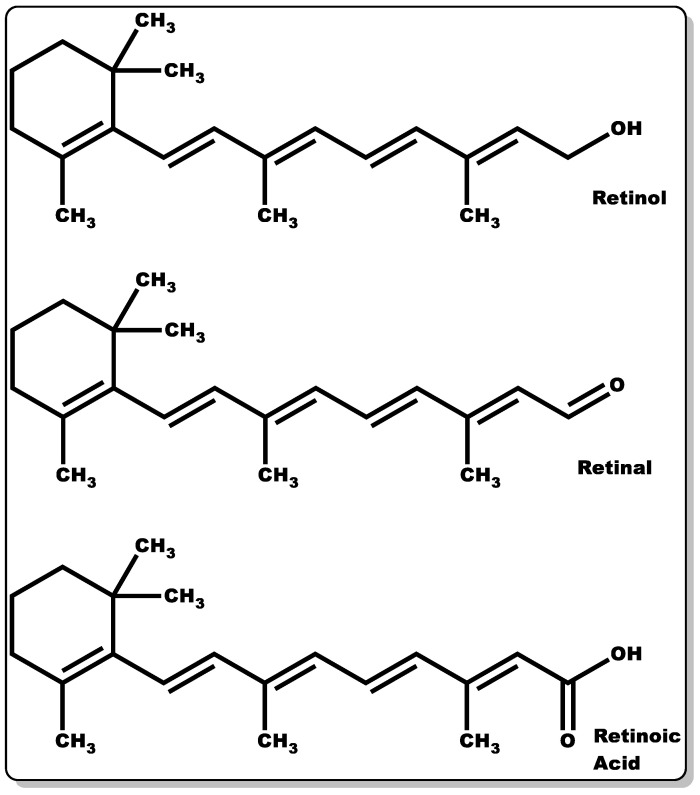
Molecular structures of retinol, retinaldehyde, and retinoic acid.

Retinoids are a group of compounds with a lipophilic non-aromatic β-ionone ring having an unsaturated isoprenoid side chain (Figure 6). This class of chemical compounds consists of retinol and its derivatives, which are known for their pharmacological and physiological roles. These include the treatment of vision impairment and skin disorders, such as photodamage, acne vulgaris, wrinkles, and psoriasis [94,95,96,97]. Furthermore, they have demonstrated efficacy in the management of other skin abnormalities, such as disordered fibrotic proliferation, including hypertrophic scars, keloids, and scleroderma [97,98]. They continue to play an essential role in efficient epithelial keratinisation through the regulation of the proliferation and differentiation of several cell types within the skin, including keratinocytes and fibroblasts [97,99]. Additionally, they have been shown to modulate gene transcription by controlling the extracellular matrix (ECM) through elevated collagen and fibronectin generation coupled with decreased collagenase activity and the recruitment of local inflammatory mechanisms to foster wound healing [97]. Moreover, retinoid offers protection against ultraviolet–B (UVB)-induced DNA damage [100,101,102,103]. Retinoids’ crucial functionality in the epithelialisation and subsequent wound healing of compromised skin tissues is well documented [97]. Retinoids can be classified into four generations [96], which are listed as follows: (i) retinol, retinaldehyde (retinal), retinoic acid (tretinoin), isotretinoin, and alitretinoin belong to the first-generation class of retinoids, (ii) etretinate and its metabolite acitretin are the second generation, and (iii) the third-generation class includes bexarotene, tazarotene, and adapalene. Finally, (iv) the fourth generation includes trifarotene. However, the focus of this review will be on the first-generation retinoids, with particular emphasis on retinol, retinal, and retinoic acid (Figure 6).

According to Törmä and co-workers, 90% of the retinoids in the skin are made up of retinyl ester, and retinol accounts for only 10% [104]. They have been reported to have a high capacity to absorb ultraviolet beam (UVB) radiation ranging from 300 to 350 nm [104]. This was demonstrated in a study by Antille and co-workers, where they investigated the skin photo-protection capacity of retinyl palmitate in the presence of high UVB radiation exposure. The outcome of the findings corroborated the photo-protection of the epidermis and anti-photocarcinogenic properties of retinyl ester [105]. 

Moreover, in a study by Pechère and co-workers where retinol and its natural derivates (retinal and retinoic acid) were tested against bacterial strains, only retinal and retinoic acid demonstrated inhibitory activity against *S. aureus* or *P. acnes*, with retinal affording more potent antibacterial activity compared to retinoic acid. The MIC of retinal against various Gram-positive bacteria (strains) is presented in Table 2 [106,107]. 

However, a recent study by Harris et al. showed that retinol can serve as a good agent in the prevention of microbial infections, particularly against *S. pyogenes*. [108]. The antimicrobial mechanism of action of retinal is thought to be the interaction of the adamantane component of retinal with the lipophilic layer of the bacterial cell membrane, thereby causing the disruption of its biosynthetic pathway [96]. Retinoic acid has been demonstrated to inhibit inflammatory reactions at the homeostasis phase by regulating the gene expression of inflammatory infiltrates and proinflammatory cytokines (tumour necrosis factor (TNF), interleukin-6 (IL-6), and nitric oxide (NO)) that could cause persistent inflammation [109]. Essentially, it can be postulated that there is a synergy in the pharmacological and physiological actions of retinol and its metabolites for wound healing; the retinal component has been proven to inhibit bacterial growth and proliferation, with retinoic acid modulating the homeostasis phase by regulating the influx of inflammatory infiltrates responsible for persistent inflammation and retinol modulating the growth factor expression essential for tissue regeneration [97,106,107,108,109].

#### 3.3.1. Retinol

Retinol was first isolated from *Scombresox saurus* liver oil by Karrer in 1931 [110] (Figure 6). The compound is naturally found in animal products, and as chemical precursors in fruits and vegetables. Retinol is a fat-soluble molecule with antioxidant and wound-healing dispositions. Retinol, in comparison to its metabolites, does not have the same profound pharmacological and physiological properties [111]. Excessive administration of retinol could lead to skin irritation, such as erythema, dryness, peeling, pruritis, and stinging/burning [112]. For retinol to exert similar pharmacological and physiological responses comparable to those of its metabolites, a higher dose of retinol may be required, which could lead to adverse effects [113]. These adverse effects could be due to the excessive stimulation of epidermal turnover and cell proliferation, leading to hyperplasia and spongiosis (localised swelling of the epidermis) [114]. The ideal concentration of retinol can cause an increase in the epidermal thickness [115], which can occur through several processes. This could occur by the upregulation of genes related to collagen type I (COL1A1) and III (COL3A1), which in turn increase the protein expression of procollagen I and III [113]. The improved collagen production can reduce fine wrinkles and scar formation [103]. Using a 1% topical retinol application, increased fibroblast growth, increased collagen synthesis, and a reduction in matrix metalloproteinases were observed, all of which counteract the effects of photoageing or natural ageing in the skin [100]. As well as having a direct effect, retinol can increase the expression of cellular retinoic acid-binding protein II (CRABPII) [115], cellular retinol-binding protein (CRBP) mRNA, and protein [113]. The skin thickening disposition of retinol was demonstrated in a study by Kang and co-workers, where they applied all-trans-retinol to the healthy human epidermis, and it fostered epidermal thickening and elevated the mRNA expression of cellular retinoic acid and retinol-binding protein [114]. In another study by Varani and co-workers, the topical administration of retinol was found to decrease matrixins expression and elevate fibroblast proliferation and the production of collagen in naturally aged skin, as performed in photoaged skin [100,111]. Matrixins, also known as matrix metalloproteinases, are enzymes capable of degrading ECM and they play vital roles in cell growth, migration, differentiation, angiogenesis, apoptosis/necrosis, and host defence [116]. Essentially, they have been reported to influence the physiological or pathological functioning of the biological system, including metastasis, inflammation, and wound healing (tissue remodelling—angiogenesis, and epithelialisation) [116,117].

Retinol deficiency can lead to a general impairment of wound healing, characterised by delayed epithelialisation [97], and can lead to abnormal epithelial keratinisation [99]. This has been proven in a rat model [118]. Steroids are known to contribute to wound healing delay. In a study by Ehrlich and co-workers, the inhibitory activity of retinol against anti-inflammatory steroids was demonstrated, which could play a profound role in the wound recovery timeframe [119].

#### 3.3.2. Retinal

Retinal was first isolated in 1934 by Wald and, in 1944, Morton suggested that the compound in question was vitamin A aldehyde, linking it to the previously recognised retinol [120] (Figure 6). Retinal is obtained by the hydrolysis of β-carotene, a retinoid precursor that is found in many fruits and vegetables. 

The antibacterial activity of retinal was demonstrated by Pechère et al. where 0.05% of retinal was topically administered against *Propionibacterium acnes* and significant bactericidal activity was observed [106]. Retinal showed significant in vitro antibacterial activity against Gram-positive bacteria; however, there was no observed activity found against Gram-negative bacteria. It is hypothesised that the antibacterial effect is, in part, due to the aldehyde group in the lateral chain [106]. 

Much of the other effects of retinal are indirect, such that the functionality is from nuclear receptor binding, hence leading to gene modulation [107].

#### 3.3.3. Retinoic Acid

**Figure 7:**
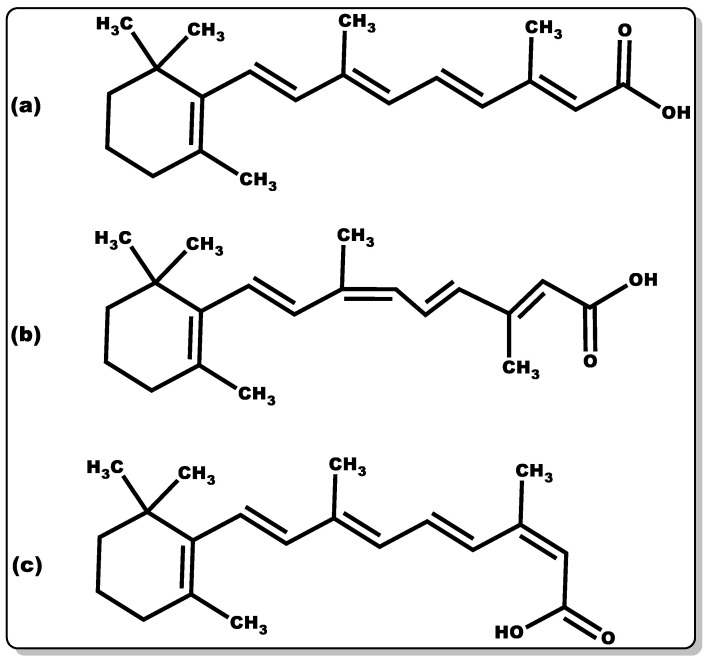
Molecular structure of retinoic acid in various isoforms: (**a**) all-trans-retinoic acid, (**b**) 9-cis-retinoic acid, and (**c**) 13-cis-retinoic acid.

Retinoic acid (Figure 6) is highly reactive and hence possesses low stability. The half-life of retinoic acid is approximately 1 h, which is in part due to CYP metabolism performing hydroxylation [121]. There are several known isoforms of retinoic acid. The most common retinoic acid with physiological activity is an all-trans-retinoic acid [122]. There are two other well-known isoforms, such as 9-cis-retinoic acid and 13-cis-retinoic acid (Figure 7). 

Before the retinoic acid can bind to the receptors, to elicit the desired effect, they must first be transported to the correct location within the cell. Cellular retinoic acid-binding proteins (CRABPs) bind to the all-trans-retinoic acid, with high affinity, and can then be transported into the nucleus [122].

Retinoic acids mainly regulate gene expression via interaction with both nuclear and cytosolic receptors [97]. There are specific retinoic acid receptors (RARs) that are important regulators for development. There are three characterised RAR-coding genes: -α, -β, and -γ, and retinoid X receptors (RXRs). These receptors are expressed in fibroblasts and keratinocytes, and the expression of these receptors is even regulated by retinoic acid [122]. Retinoic acid can block collagenase activity, the enzymes that break down collagen, hence preventing collagen degradation [113]. Retinoic acid can regulate gene expression in both the epidermis and dermis. The genes are modulated concerning translation, transcription factors, RNA metabolism, receptor expression, and apoptosis. All-trans-retinoic acid is used in the treatment of skin cancer and acute promyelocytic leukaemia (APL). Conversely, a deficiency of retinoic acid has been associated with cancer progression and various dermatological diseases [122].

Similar to retinol, retinoic acid is also commonly used to treat acne and wrinkles/ageing [122]. This is due to the impact of retinoic acid on increased epithelial cell differentiation and proliferation, as well as the proliferation of keratinocytes and fibroblasts [97].

All trans-retinoic acid has been shown to have fungistatic effects, which can be used for psoriasis patients possessing a predisposition to fungal infections [123].

### 3.4. Vitamin C

**Figure 8:**
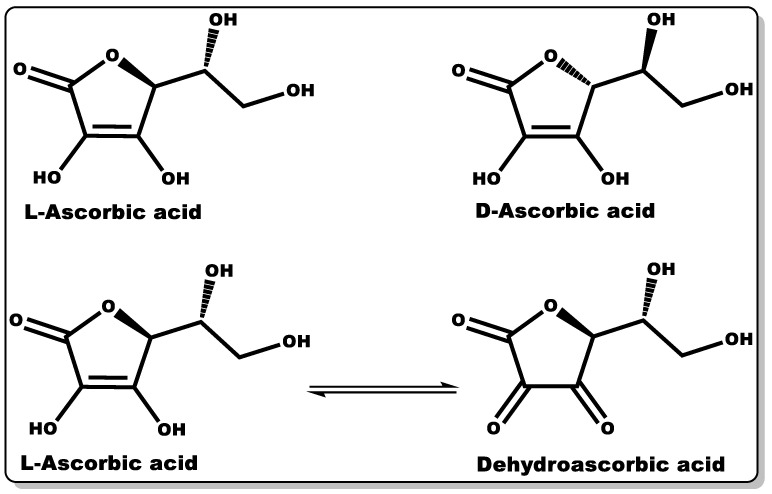
Molecular structures of ascorbic acid and its oxidised form.

Vitamin C (VTC, also known as ascorbic acid (Figure 8)) is a hydrophilic molecule with potent pharmacological and physiological activities, including antioxidant, anti-inflammatory, antimicrobial, and wound healing efficacy [124,125,126,127]. Plant sources are known to possess an abundant amount of VTC, including vegetables and fruits. Traditionally, they have been explored as antioxidants in food supplements, preservatives [128], and the management or prevention of scurvy [125]. The immunomodulatory activity of VTC in influencing the signalling pathway for cell differentiation and proliferation is well documented [129,130,131]. VTC is a gluconic acid lactone obtained from glucuronic acid and hydrophilic keto-lactone having two ionisable hydroxyl moieties [132]. VTC mostly exists in two equal enantiomers, including D-ascorbic acid and L-ascorbic acid which are mutually interchangeable [132]; however, the most common and bioactive isomer of VTC is L-ascorbic acid.

Dehydroascorbic acid (DHAA) is an oxidised form of ascorbic acid (AA), and it can be converted to AA in the presence of a reducing agent [132]. AA and DHAA have been applied as active ingredients in cosmetic formulations and antimicrobial agents in pharmaceutical products [133,134,135,136,137], especially in skin tanning and the treatment or prevention of gingivitis, respectively [133,134,135]. Numerous studies have demonstrated the antimicrobial activity of VTC against both Gram-negative and Gram-positive bacteria, including *S. mutans*, *P. gingivalis*, *S. aureus*, *H. pylori*, *B. subtilis*, and *M. tuberculosis*, and fungi including *C. albicans*, *Aspergillus niger*, and *A. flavus* [138,139,140,141,142,143]. In a dose-dependent study by Verghese and co-workers, VTC was found to inhibit the growth of uropathogenic *Escherichia coli* and *K. pneumoniae* at a MIC value of 1% [138]. Moreover, Isela et al. demonstrated MIC values of VTC against *S. mutans*, *S. aureus*, *P. gingivalis*, *C. albicans*, and *E. faecalis* and their biofilms of 1% and 2%, respectively [141]. According to Mousavi et al., the MIC of VTC against *C. jejuni*-infected mice was found to be 0.1409% at pH 7.3 [126]. Moreover, a number of studies have demonstrated the disruption of bacterial biofilms at low VTC concentrations [142,144]. The prevention or inhibition of bacterial biofilm formation by VTC is attributed to its bacterial anti-quorum-sensing properties and the disruption of extracellular polymeric substance (EPS) biosynthesis. The EPS matrix is mainly made up of polysaccharides, proteins, and extracellular DNA, which affords defence to bacterial biofilms against host immunity and antibiotics from attacking bacteria [29,126]. This antibiofilm-formation property of VTC could be explored with other molecules (antibiotics) that can directly attack and eliminate AMR recalcitrant planktonic bacteria, including *P. aeruginosa* and MRSA [126]. 

VTC is innocuous against skin cells, making it suitable for topical cosmetical formulations [136,137]. The physiological role of VTC is imperative due to its crucial activity in skin fibroblast growth and migration, as well as the production of collagen and elastin, which are vital for wound healing or contraction [145,146,147]. It also possesses the capability to prevent changes associated with photoageing [130,131,147]. The wound healing efficacy of VTC was proven by Bikkera et al., where they investigated the impact of AA on wound healing in surgical patients, and it was found that AA deficiency impairs wound healing [124]. The wound care efficacy of VTC is attributed to its antioxidant, anti-inflammatory, antimicrobial, and collagen synthesis properties [124,125,127,147]. Several studies have shown the anti-inflammatory activity of VTC, and this follows its capacity for the downregulation of proinflammatory cytokines causing persistent inflammation [126,148].

A report by Lykkesfeldt et al. demonstrated the capability of VTC in the regeneration of vitamin E (tocopherols) from its oxidised form (tocopheroxyl radical), thereby affording VTC to indirectly inhibit lipid peroxidation [125]. Moreover, the combination of VTC and vitamin E has been demonstrated to afford maximum photoprotection of the skin, thereby limiting photoageing [149,150,151,152,153]. 

### 3.5. Vitamins E

**Figure 9:**
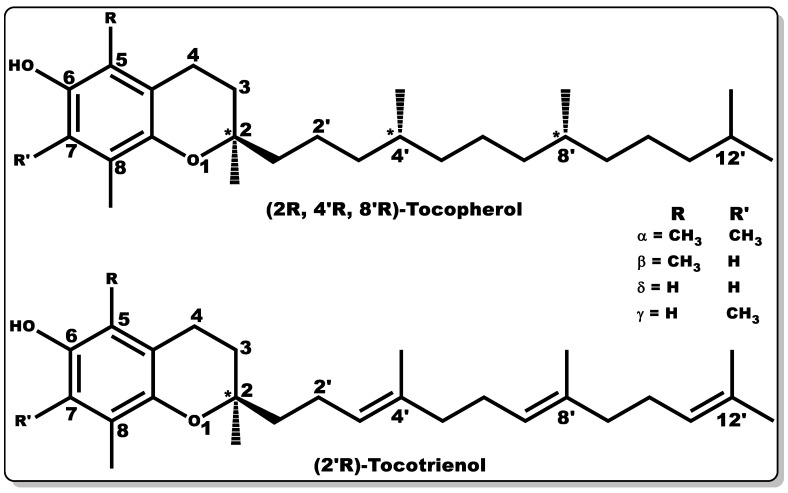
Molecular structures of tocochromanols (tocopherol and tocotrienol). Various isoforms of tocochromanols vary at position 5 or 7 (R or R’) of the chromanol ring with either -H or -CH_3_ moieties. In addition, the side chain of both tocochromanols differ, with tocopherol having a saturated chain while tocotrienol possess unsaturated side chain (double bond) at positions 3′, 7′, and 11′.

Vitamin E (VTE, also known as tocochromanol) is composed of two major hydrophobic low-molecular-weight compounds grouped as tocopherols and tocotrienols. Tocopherols and tocotrienols are structurally identical with their chromanol rings (Figure 9), but have differences in their side chain, with the former having a long, saturated chain (phytyl) and the latter showing an unsaturated chain (farnesyl) with double bonds at positions 3′, 7′, and 11′, [154]. 

Both tocochromanols have eight subgroups, with each group accounting for four “isomers” each, existing as alpha (α), beta (β), delta (δ), and gamma (γ) [154,155,156]. VTE possesses anti-inflammatory, antioxidant, antibacterial, and wound-healing properties [154,156,157,158,159,160,161]. Furthermore, vitamin E is capable of preventing biofilm formation [162]. VTE is mostly obtained from natural sources, including plant seeds, nuts, corn, soybean, fruits, and vegetables [155,156,163,164,165]. α–Tocopherol is the major vitamin E component in humans with bioactivity [156,166,167], and its regulation of metabolic processes has been well documented [156,157,159,160]. α–Tocopherol has been used as a dietary supplement and as a component of skincare formulations [168,169]. 

**Figure 10:**
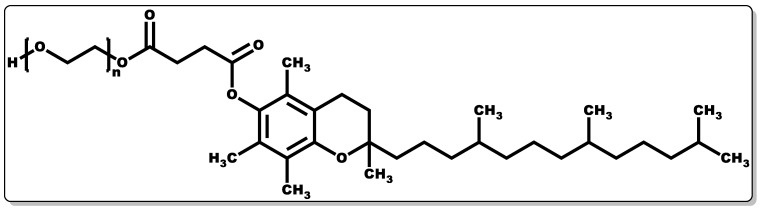
Structure of α-tocopheryl polyethene glycol 1000 succinate.

Tocopherols are lipid-soluble molecules with ease of skin penetrability due to their low molecular weight, and they have been applied when alone or in combination with other molecules. Their antioxidant activity is devoid of skin irritation and they are capable of inhibiting allergic epidermal reactions, making them suitable for topical application [131,170,171]. In a study by Kuriyama et al., the topical administration of tocopherol was found to inhibit the irritation and allergic reaction often associated with contact dermatitis by regulating the keratinocytes [170]. Its antioxidant activity functions by transferring hydrogen to free radicals, including peroxyl, oxygen, and superoxide anions, thereby scavenging the radicals, affording protection to polyunsaturated fatty acids (PUFAs) from oxidation, inhibition of lipid peroxidation, and reduction of the skin ageing rate [171,172]. Moreover, tocopherol has been reported to have the capacity to regulate T-cell proliferation and interleukin-2 generation [161,173,174,175]. Furthermore, it has been shown to serve as an enzyme activity modulator, including protein kinase C (PKC), responsible for cell-mediated immune responses and cell proliferation, such as smooth muscle growth. It plays a role in the deactivation of PKC by inhibiting smooth muscle growth [174,176,177]. Tocopherols have been shown to possess potent biological activity in preventing infectious diseases [175]. Tocopherol by itself or when combined with antibiotics has demonstrated antibacterial activity [161,178]; however, its interaction with other molecules has been proven to have broad applicability [179,180]. To obtain the water-soluble or amphiphilic form of tocopherol, the esterification of the tocopherol derivative (D-α-tocopheryl succinate) with polyethene glycol 1000 results in D-α-tocopheryl polyethene glycol 1000 succinate (TPGS) (Figure 10) [181]. TPGS, a hydrophilic form of tocopherol, is made up of a lipophilic α-tocopherol and a hydrophilic PEG chain [181]. 

Studies have shown that TPGS has high bioavailability in comparison to hydrophilic tocopherol formulations in children with chronic cholestasis, indicating the potential of TPGS to serve as an alternative to tocopherol in order to avoid the injection of vitamin E formulations in chronic cholestasis [181,182]. TPGS is generally classified by USFDA as a safe substance, which has given it applicability in the pharmaceutical industry as an adjuvant to enhance drug molecules’ solubility, absorptivity, stability, and bioavailability [181]. A number of studies have shown the improvement in the oral absorptivity of vancomycin hydrochloride and talinolol in animals when in formulation with TPGS [183,184]. TPGS has been successfully used as a nano-vehicle for the delivery of drug molecules with low solubility and poor permeability [179,180]. A known example is cisplatin, a potent antineoplastic agent with poor hydrophilicity; however, upon combination with TPGS, there was a remarkable improvement in its physicochemical disposition [179,180]. TPGS has been reported to possess antitumorigenic activity when alone and in combination with other drug molecules, and this is evidenced by its improved pharmacological response in formulation with cisplatin [179,180]. Vitamin E or TPGS have been reported to synergistically elicit antibacterial activity when combined with other molecules (e.g., antibiotics) by downregulating efflux pump gene expression, leading to the lowering of the bacterial efflux pump activity, allowing the effective dose of antibiotics to reach the target bacterial cells [161,178,185]. Moreover, vitamin E or TPGS can enhance the penetration of antibiotics into bacterial cells, making them a suitable pharmaceutical adjuvant for antibiotics [186]. There is growing research demonstrating that other forms of VTE possess similar or superior biological activity in comparison to α-tocopherol [187]. In particular, the superior functionality of tocotrienols results in more effective penetration and distribution in the lipid layers of the cell membrane due to their unsaturated side chains having a higher affinity for the saturated lipid layers of biological tissues, including the brain and liver [187,188,189]. Tocopherols and tocotrienols (Figure 9) only differ in their side chains, with the latter having double bonds (unsaturated) at positions 3′, 7′, and 11′, as mentioned earlier. However, both have four different forms each, often classified as α, β, δ, and γ [189,190]. For instance, tocotrienols have been reported to exhibit superior antioxidant, analgesic, anti-inflammatory, antibacterial, anti-cancer, neuroprotective, and cholesterol modulation properties in comparison to those demonstrated by tocopherols [154,187,189]. Studies by Pearce and co-workers demonstrated the efficacy of tocotrienol at micromolar concentrations in inhibiting the enzyme in the liver (HMG-CoA reductase) responsible for the synthesis of cholesterol [191,192]. 

Overall, both tocopherols and tocotrienols possess significant biological activities, including antioxidant, anti-inflammatory, and antibacterial dispositions, which could be responsible for their wound healing efficacy. As demonstrated by several researchers, the wound healing efficacy of tocochromanols, when combined with antibiotics, is quite profound for preclinical data with clinical potential in humans [154,193,194]. In many studies, the oral and topical administration of tocochromanols was found to elicit wound healing efficacy. All of the findings regarding tocochromanols, when alone or in formulation with other molecules, demonstrated them fostering angiogenesis, epithelisation, granulation, and collagen production, accounting for rapid wound contraction and tissue regeneration [158,195,196,197,198,199]. 

### 3.6. Chitosan

**Figure 11:**
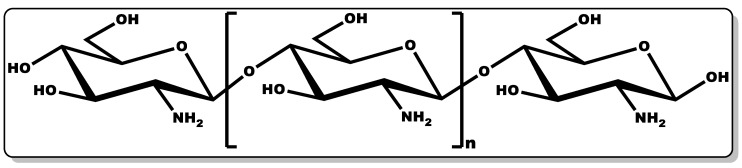
Structure of chitosan.

Chitosan (CTN, Figure 11) is a biocompatible linear amino polysaccharide consisting of glucosamine and N-acetyl glucosamine units connected through β-(1→4) glycosidic bonds [7,200]. CTN is obtained from chitin, which is mostly found in crustaceans and shellfish. The versatility of CTN has earned it applicability in several industries, including medicine, pharmaceutical, cosmetics, agrochemistry, food, and beverage [7,200]. 

CTN pharmaceutical and medical applications have demonstrated its pharmacological and physiological roles, including its antioxidant, anti-inflammatory, antimicrobial, and wound healing efficacy [7,201,202,203,204,205,206]. Its potency in fostering chronic wound healing has been explored in various forms, including as powders, hydrogels, sponges, nanoparticles, bandages, and films [7,206]. Several authors have reported the antimicrobial and wound healing efficacy of CTN when incorporated in bandages alone or in combination with antibiotics [206,207,208,209]. This was proven when bandages containing only CTN were administered on bleeding wounds, leading to the rapid inhibition of haemorrhaging [7,206,207,208,209,210]. This observation could be attributed to the positively charged polysaccharide amine of CTN attracting negatively charged red blood cells (RBC), fostering blood clotting through the electrostatic interaction of the CTN and RBC [7,206,207,208,209]. In a study by Nimal et al., they demonstrated the remarkable efficacy of CTN bandages containing antibiotics with sustained release of the antibiotics for two weeks, leading to a significant reduction in the bacterial loads of the various polymicrobial cultures tested, including *C. albicans*, *E. coli*, and *S. aureus* [201]. In another study by Marangon et al., it was further established that the incorporation of CTN with rhamnolipid not only improved the antibacterial activity of the antibiotic agent against diverse strains of *Staphylococcus*, but also stabilised the CTN, showing the effective synergy between the two molecules [202]. Furthermore, studies by several authors have demonstrated the effectiveness of CTN in promoting tissue remodelling, with a reduction of scar tissue and an increase in the wound healing efficacy [7,203,204,205,206,210,211]. This is evidenced by a study conducted by Baxter and co-workers, in which a chitosan dressing was applied to a third-degree burn (mice model) leading to wound contraction [203]. The chitosan modulation of transforming growth factor-β1 (TGF-β1) and collagen III deposition in the wounds facilitated tissue remodelling and a subsequent reduction in TGF-β1, preventing the formation of a scar at the wound site. This is coupled with the recruitment of fibroblasts and the inhibition of inflammatory cytokines release, affording limited-pain wound healing [203]. Moreover, antimicrobial and wound healing study conducted by Dai et al. on mice infected burn, demonstrated the efficacy of CTN in the rapid bactericidal activity against pathogenic bacteria whilst promoting wound recovery [210]. 

CTN works by modulating the various cellular processes involved in wound healing by reducing the microbial loads and regulating growth factor expression (such as epidermal growth factor and TGF-β1) during wound healing phases [201,202,203,204]. In chronic wounds, CTN is thought to reduce the bacterial load by inhibiting or eliminating polymicrobial growth in infectious wounds. This antibacterial activity of CTN is achieved when the positively charged component of CTN interfaces with the negatively charged component of the bacterial cell membrane [201,202,203,204]. This coherence results in the inhibition of the bacterial cell membrane’s protein biosynthesis and translation. CTN is efficacious against both Gram-positive and Gram-negative bacteria [204]. However, it is more potent against Gram-negative bacteria, and this is adduced to the highly negatively charged envelope that the cell wall of Gram-negative bacteria possesses, which has a greater affinity for the positively charged polysaccharide amine group of CTN [204]. Furthermore, it fosters the efficient migration of neutrophils with the subsequent proliferation of fibroblasts. This is followed by its facilitation of macrophages and neutrophil infiltration and migration at the wound site, leading to the elimination of extraneous matters and promotion of granulation/fibrous tissue and re-epithelialisation. Its antimicrobial characteristics make it ideal for the prevention of wounds’ microbial infection or the inhibition of microbial growth in infected wounds [7,201,202,204,210]. Moreover, the tissue regeneration capacity of CTN is essential for wound contraction and re-epithelialisation [7,203,204,205,206,207,210]. CTN has been explored alone and in combination with other molecules or as a pharmaceutical excipient [7,200,212], and it is well tolerated and biocompatible [174,213,214]. CTN could serve as an ideal gelling agent and adjuvant for the controlled release of active ingredients for topical cosmetic formulations due to its biocompatibility, biodegradability, and compatibility with other cosmetic active ingredients, including vitamins [7,207,215,216].

### 3.7. Aloe vera

*Aloe vera*, from the Liliaceae family, has proven pharmacological activities against dry skin, burns, acne, psoriasis, and wounds [217,218,219,220]. It has been well applied in several industrial applications, including cosmetics, food, and beverages. Its use in cosmetic topical application could be attributed to its moisturising and soothing effect [221,222]. The phytoconstituents of *Aloe vera* include water, vitamins (A–C and E), minerals (Na, K, Fe, and Zn), phenolics, and amino acids (folic acid). Interestingly, these components have been demonstrated to possess therapeutic activity, such as antimicrobial, anti-inflammatory, and wound healing [217,218,219,220]. Reports have exhibited the antibacterial disposition of *Aloe vera* against both Gram-positive and Gram-negative bacteria, with MIC of ≤ 0.000625% for *Pseudomonas aeruginosa*, *Bacillus subtilis,* and ≤0.005% for *Staphylococcus aureus* [223]. In another study by Goudarzi et al., *Aloe vera* was found to be efficacious against *P*. aeruginosa strains from burn wounds with an MIC value of 0.02% [224]. The antibacterial activity of *A. vera* may be due to its anthraquinone phytoconstituents [223,224]. Furthermore, the anti-inflammatory and wound healing efficacy of *A. vera* has been shown by many reports [219,220,225]. The preventive and healing effect of *A. vera* against pressure ulcers was demonstrated by Hekmatpou and co-workers, where they carried out a randomised triple-blind clinical trial, and it was observed that *A. vera* was capable of preventing or fostering the healing of pressure ulcers by modulating the wound’s temperature, non-blanchable redness, swelling, and pain [220]. Numerous studies have demonstrated the efficacy of *A.* *vera* in the modulation of proinflammatory cytokine gene expression, a known promoter of IL-6, NO, causing persistent inflammation [219,220,225]. This inhibition has been attributed to reduced inflammatory reaction and rapid wound healing [219,220]. Moreover, *A. vera* has been shown to possess tissue regeneration disposition by fostering fibroblast proliferation with collagen biosynthesis [220].

### 3.8. Cinnamaldehyde

**Figure 12:**
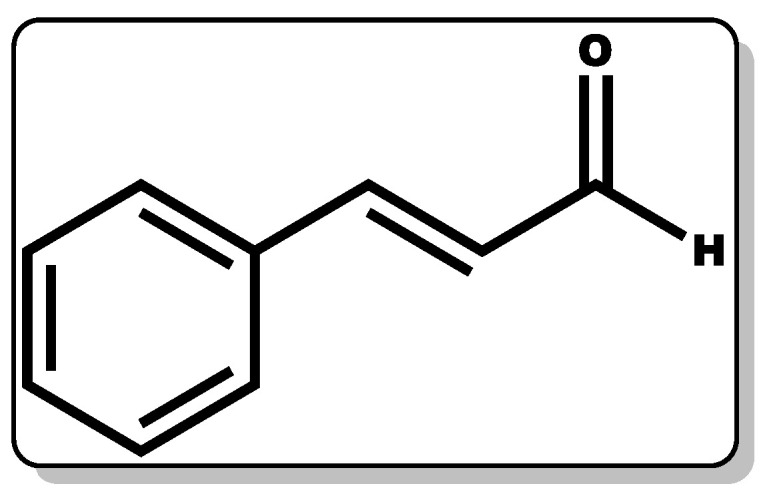
Molecular structure of cinnamaldehyde.

Cinnamaldehyde (CME, Figure 12) is a phenylpropanoid molecule obtained from the bark of cinnamon trees with proven therapeutic action, including antimicrobial, anti-inflammatory, and wound healing efficacy [38,226,227,228]. 

It has been found to be useful in the beverage, cosmetic, and agrochemical industries [38,229,230]. CME has exhibited activity in the inhibition or elimination of pathogenic fungi, including *Candida albicans* and *Aspergillus flavus* [227,228]. Moreover, this compound has been shown to repel insects, kill or inhibit certain bacterial growth, and prevent biofilm formation [29,230]. Several studies have demonstrated the antimicrobial properties of cinnamaldehyde, including pathogenic Gram-positive and Gram-negative bacteria, such as *P. aeruginosa*, *E. coli*, and *S. aureus* [29,231]. According to Ramasamy and co-workers, the efficacy of CME against *P. aeruginosa*, *E. coli*, and *S. aureus* is limited at concentrations ranging from 0.0005% to 0.025%; however, on incorporation with nanoparticles, the MIC and MBC were greatly improved, inferring the synergistic disposition between CME and other molecules. In addition, in a study conducted by Topa et al., it was reported that CME with MIC (0.16%) was found to elicit bacteriostatic action against *P. aeruginosa* [29]. Another study by Utchariyakiat and co-workers showed that the MIC of CME against *P. aeruginosa* ranged from 0.0562% to 0.225% [232]. A recent study by Pereira and co-workers showed that CME is potent against *E. coli* at a MIC of 0.078% [233]. In a similar study, it was established that CME was well tolerated by human epithelial cells [233]. The antimicrobial action of CME may be by disrupting the cellular homeostasis of the bacterial cell membrane, thereby impeding its growth [233]. Moreover, CME has been shown to possess an anti-inflammatory disposition, which is essential for wound management [234]. The antibacterial and anti-inflammatory dispositions of CME are useful in the management of wounds due to its capacity to eliminate or prevent bacterial biofilms (*P. aeruginosa*) and its reduction of the inflammatory reaction by inhibiting high-influx of inflammatory infiltrates. Moreover, numerous reports have demonstrated that CME is capable of accelerating collagen production and the induction of mammalian endothelial cell growth, which is crucial for wound healing [226,235,236]. This was demonstrated in a study by Ferro et al. where CME was tested against *P. aeruginosa*-infected mice skin wounds, and it was observed that the bacterium metabolic rate and its ability to cause biofilm formation was reduced at sub-inhibitory concentrations of CME. Furthermore, routine topical administration of CME was reported to have lowered the bacterium bioburden of the mice’s skin wounds with rapid wound contraction and healing. Further analysis showed that the CME-treated wound samples had lower interleukin-17, vascular endothelial growth factor, and nitric oxide levels compared to the untreated wound samples [236]. The modulation of these inflammatory infiltrates by CME may have contributed to its wound healing action.

## 4. Benefits and Limitations of Antibiotics-Free Compounds for Chronic Wounds

Antibiotics-free compounds are capable of accelerating chronic wound healing when administered at low concentrations. They not only offer antibacterial efficacy, but also influence each stage of the cellular and morphological events of the wounds, including the regulation of the inflammatory, proliferative, and tissue-remodelling phases. The many benefits and potential limitations associated with the discussed compounds are stated in Table 3. The pharmacological and physiological activities of these compounds are influenced by their concentration, temperature, formulation, presence of organic matter, and contact time [9].

## 5. Challenges for Drug Delivery to Chronic Wounds 

The current treatment modalities for chronic wounds are hampered by the wound microenvironment having polymicrobial growth and biofilm formation, making the delivery of therapeutic doses of antibiotics at the target site difficult. This limitation may account for the persistent inflammation and delayed wound healing often reported in infectious wounds. Bacterial biofilms have been shown to afford a protective shield to bacteria through their EPS, making them evasive to both antibiotics and host immunity [9,10]. Some of the conventional approaches that have been explored in the management of CWs include debridement to remove necrotic cells, therapeutic cleansing using a biocide to reduce microbial bioburden, wound dressing, and antibiotics to eliminate bacterial loads. However, these methods are not always helpful in the management of CWs, and this is complicated by the poor blood circulation in most CWs, making the systemic delivery of antibiotics at infectious wound sites difficult. Furthermore, the use of wound dressings impregnated with antiseptic agents could potentially increase the application duration, but they neither control the release nor improve the penetrability of antibacterial agents. Recently, more advanced carriers have been proposed for the delivery of antimicrobial compounds, including fibres, microneedles, particulates, and vesicular carriers [10]. The use of fibres impregnated with antibiotics having sizes ranging from nm to µm, usually obtained by electrospinning, have been explored. Fibres impregnated with antibiotics have been demonstrated to circumvent CW barriers due to their fibrous morphology ability, which mimics the human extracellular matrix, fostering cell adhesion with subsequent gas exchange, the inhibition of microbial infiltrates, and modulation of a moist environment [10,243,244]. Chitosan has been seen as an ideal polymeric material for incorporation with antibiotics, and this may be attributed to its desirable physicochemical and antibacterial properties [202,244]. Microneedles incorporated with films coated with antibacterial agents have been demonstrated as a potential approach for CW treatment. This is due to their pain-free penetration of the outer layers of the skin because of their miniature size [10,245]. The size dynamics of nanomaterials is an attractive feature compelling their utilisation for the treatment of infectious wounds. Nanoparticles with sizes ranging from 1 to 100 nm have been proven to circumvent the barriers often posed by bacterial biofilms due to their small size, making them suitable candidates for carrying antimicrobial agents with improved antibacterial actions [59,67,246]. Vesicles, such as TPGS and liposomes, with hydrophilic and lipophilic phases have been explored as potential drug carriers. They may be incorporated with hydrophobic or water-soluble drug molecules due to their amphiphilic properties improving their versatility. Moreover, they are a good penetration enhancer and are capable of disrupting bacterial biofilms. This is often achieved due to their ability to potentially fuse with biological membranes, resulting in improved intracellular drug delivery with enhanced therapeutic efficacy [10,181,247]. These advanced modalities may be capable of circumventing the CW barrier, including bacterial biofilms. However, further investigation of their applicability, needs to be conducted, including their short and long-term safety. 

## 6. Conclusions

Several antibiotics-free compounds, including curcumin, *Aloe vera*, polyhexanide, cinnamaldehyde, retinoids, ascorbate, tocochromanols, and chitosan, when alone or in formulation with other molecules (antibiotics) have pharmacological and physiological roles in wound healing. Vitamin A (retinoids), vitamin C (ascorbic acid), and vitamin E (tocochromanol) are low-molecular-weight compounds with potent dermatological, pharmacological, and physiological activities. These vitamins are known for their synergistic disposition when combined in formulations. For instance, retinoids and ascorbic acid have shown increased production of collagen, which is pivotal for wound healing and tissue regeneration. Moreover, ascorbic acid (AA) has been proven to be capable of regenerating tocochromanol (tocopherol) from its radical form (tocopheryl), thereby enabling AA to inhibit lipid peroxidation [125]. Their low molecular weight is also advantageous, as their ease of skin penetration can be effectively utilised for topical dermatological applications. The biological activity of these compounds have been attributed to their antioxidant, antibacterial, anti-inflammatory, and wound healing efficacy, which has been proven to modulate the processes involved in wound healing, including inflammation, proliferation (neo-angiogenesis, granulation, and re-epithelialisation), debridement, and maturation. Some of these molecules have been shown to have activity against antimicrobial-resistant microbes and biofilms. Moreover, the combination of lipophilic and hydrophilic vitamins has been proven to have synergistic antimicrobial and dermatological properties. Interestingly, retinol and its natural derivatives have the potential for eliciting therapeutic action at every stage of wound healing with retinaldehyde, capable of exhibiting antibacterial activity against certain bacteria and retinoic acid, fostering the modulation of proinflammatory cytokines, and retinol, regulating growth factor expression necessary for tissue regeneration. Additionally, curcumin and its degradation products have biological activity that might be useful for chronic wound management. In general, a number of the considered compounds have a similar mechanism of action (MOA) in their management of wound healing. Chronic wound microenvironments exist in a cascade involving microbial infection, persistent inflammation, and impaired tissue. These infections are recalcitrant to antibiotics by the shield afforded to them by bacterial biofilms with the pH change of the CW microenvironment from slightly acidic to alkaline, which is known to promote polymicrobial colonisation and proliferation. This then fosters the persistent inflammation of CWs by promoting high influx of inflammatory infiltrates and the impairment of the tissue around the wound’s site. The wound healing action of these compounds relies on interfering with each phase of the CWs. For instance, it is postulated that the phenolic components in some of the antibiotic-free compounds are responsible for their antimicrobial activity [38,41,96]. It has been proposed that the bacteriostatic or bactericidal actions of these compounds act by altering the bacterial cell structure, preventing cell division, membrane porins, motility, and the formation of the bacterial biofilm [38,41]. In addition, they inhibit the inflammatory reaction at the homeostasis phase of the wounds by decreasing the excessive influx of inflammatory infiltrates (tumour necrosis factor (TNF), interleukin-6 or 17, and nitric oxide (NO)), causing persistent inflammation [2,38,109]. Moreover, these compounds elicit collagen biosynthesis, which is necessary for tissue regeneration.

Antibiotics-free compounds are advantageous in the management of chronic wounds as they are capable of regulating every stage of CWs, as opposed to antibiotics, which reduce the bacteria load with minimal interference concerning tissue remodelling. Moreover, some of these compounds can be employed as prophylactic agent in the prevention of bacterial biofilms or used in synergy with antibiotics in the elimination of bacteria. Overall, these compounds are suitable at a certain permissible limit, as any concentration above the standard threshold can result in adverse reactions, including proinflammation, cytotoxicity, and delayed wound healing.

## Figures and Tables

**Table 1:** Minimum inhibitory concentration (MIC) of curcumin and PHMB on various species of bacteria [42,76].

**Curcumin (MIC Values in %)**									
*S. aureus*	*P. gingivalis*	*E. coli*	*S. epidermidis*	*P. aeruginosa*		*S. mutans*	*P. mirabilis*	*S. marcescens*	*B. subtilis*
0.0188	0.0125	0.0192	0.0175	0.0192		0.0175	0.0192	0.0384	0.0100
**PHMB (MIC Values in %)**									
*S. aureus*	*P. gingivalis*	*E. coli*	*S. epidermidis*	*P. aeruginosa*	*M. luteus*	*M. smegmatis*	*S. enterica typh*	*B. subtilis*	*S. griseus*
0.0002	0.0010	0.0002	0.0001	0.0010	0.0010	0.0012	0.0004	0.0005	0.0005

**Table 2:** Minimum inhibitory concentration (MIC) of retinoids against various strains of Gram-positive bacteria [106,107]. Methicillin-sensitive *S. aureus* (MSSA) and methicillin-resistant *S. aureus* (MSSA). Not active (NA).

*P. acne* Strains				*S. aureus* Strains	
	CIP179	CIP53119	CIP53117	MSSA	MRSA
Retinal	0.0004%	0.0004%	0.0008%	0.0008%	0.0004%
Retinoic acid	0.0128%			NA	

**Table 3:** The advantages, disadvantages, and challenges of each compound in treating chronic wounds.

Compounds	Benefits	Limitations
Curcumin	- Antibacterial and wound-healing agent [42,56,70] - Modulation of cellular and molecular pathways, including the regulation of inflammation and tissue regeneration [70]	- Sparing to low aqueous solubility [64] - Low stability due to photo- and pH sensitivity [65]
Polyhexanide	- Efficacious antibacterial and wound-healing agents [84,85]. - Potent and well tolerated in wounds at low concentrations (0.02–0.5%) with potential to induce re-epithelialisation [89,237,238]	- Carcinogenic at high concentrations [43,89,93]. - Its antibacterial activity may be influenced by pH [239].
Retinol, Retinaldehyde, Retinoic acid	- Antibacterial and tissue regeneration agents [96,106,107,111]	- Limited antibacterial activities [106,107] - Sparing to low aqueous solubility [111] - Toxic at high concentrations [240]
Ascorbic acid	- Antibacterial and tissue regeneration agent - High aqueous solubility [141,147]	- Low photostability
Tocochromanols	- Antibacterial and tissue regeneration agent - Excellent amphiphilic characteristics when modified (TPGS) - Penetration enhancer [TPGS] making it a suitable drug carrier and delivery agent [161,181]	- Limited antibacterial activities - Sparing to low aqueous solubility when present in its pristine form [181]
Chitosan	- Ideal antibacterial and tissue regeneration agent - Excellent drug delivery agent for wound healing [7,201,202,203,204,205,206]	- Sparing to low aqueous solubility when present in its pristine form [7] - Toxic at high concentrations [204]
*Aloe vera*	- Antibacterial and wound healing efficacy [220,221,222,223]	- May cause contact dermatitis with mild redness and itching [241]
Cinnamaldehyde	- Potent antibacterial and tissue regeneration agent [29,233]	- Carcinogenic at high concentrations. - Sparing to low aqueous solubility [233,242]
